# Current status of peptide receptor radionuclide therapy in grade 1 and 2 gastroenteropancreatic neuroendocrine tumours

**DOI:** 10.1111/jne.13469

**Published:** 2024-11-20

**Authors:** Jelka Kuiper, Eline Zoetelief, Tessa Brabander, Wouter W. de Herder, Johannes Hofland

**Affiliations:** ^1^ Department of Internal Medicine, Section of Endocrinology Erasmus MC Cancer Institute Rotterdam The Netherlands; ^2^ Department of Radiology & Nuclear Medicine Erasmus MC Cancer Institute Rotterdam The Netherlands

**Keywords:** ^177^Lu‐DOTATATE, grade 1 and 2 neuroendocrine tumours, gastroenteropancreatic neuroendocrine tumours (GEP NET), low‐grade and intermediate‐grade neuroendocrine tumours, peptide receptor radionuclide therapy (PRRT)

## Abstract

Peptide receptor radionuclide therapy (PRRT) using [^177^Lu‐DOTA^0^,Tyr^3^]octreotate (^177^Lu‐DOTATATE) represents an established treatment modality for somatostatin receptor‐positive, locally advanced or metastatic gastroenteropancreatic neuroendocrine tumours (GEP NET) of grade 1 or 2. The studies have demonstrated that four cycles of PRRT with ^177^Lu‐DOTATATE prolongs progression‐free survival and preserves quality of life, in patients with grade 1 and 2 advanced GEP NET. Notably, first‐line PRRT using ^177^Lu‐DOTATATE in grade 2 and 3 GEP NET patients has also shown efficacy and safety. Furthermore, PRRT can ameliorate symptoms in patients with NET‐associated functioning syndromes. Although various studies have explored alternative radionuclides for PRRT, none currently meet the criteria for routine clinical implementation. Ongoing research aims to further enhance PRRT, and the results from large clinical trials comparing PRRT with other NET treatments are anticipated, potentially leading to significant modifications in NET treatment strategies and PRRT protocols. The results of these studies are likely to help address existing knowledge gaps in the coming years. This review describes the clinical practice, recent developments and future treatment options of PRRT in patients with grade 1 and 2 GEP NET.

## 1 INTRODUCTION

Neuroendocrine neoplasms (NEN) are a group of epithelial neoplasms derived from the diffuse neuroendocrine system, whose incidence and prevalence has steadily increased over the past decades.^1^, ^2^, ^3^, ^4^ Despite improvements in early diagnosis, pathological classifications and treatment options, survival and quality of life (QoL) of NEN patients remains impaired. Based on histology, NEN can be divided into well‐differentiated neoplasms, termed neuroendocrine tumours (NET) and poorly differentiated neoplasms, known as neuroendocrine carcinomas. NET are graded based on the proliferation by mitotic rate and antigen Kiel 67 (Ki67) labelling into grade 1 (G1), grade 2 (G2) or grade 3 (G3), corresponding to low‐grade, intermediate‐grade and high‐grade categories, respectively.^5^ NET most commonly arise from neuroendocrine cells in the intestinal tract, bronchopulmonary tract or pancreas from neuroendocrine (progenitor) cells.^1^, ^2^ Similar to other NET, gastroenteropancreatic NET (GEP NET) are by definition tumours with malignant potential. Unfortunately, patients are on average diagnosed with a NET 2–3 years after the start of symptoms, leading to the majority of patients presenting at an advanced stage of disease when complete cure is no longer possible.^3^ Consequently, 57%–63% of all GEP NET patients die because of NET specific causes.^6^, ^7^ Patients with metastatic GEP NET G1 and G2 have a 5‐year overall survival (OS) of 47% and 38%, respectively.^8^ NET can secrete hormonally active substances, leading to NET associated functioning syndromes, which develop in 25%–30% of GEP NET patients.^9^ The most common functioning syndrome is the carcinoid syndrome, primarily caused by serotonin and tachykinin overproduction. It is characterised by increased bowel movements, vasoactive flushes, bronchospasms and long‐term fibrotic changes in the mesentery and cardiac valves.^10^, ^11^ Other hormonal syndromes include insulinoma, gastrinoma, glucagonoma, vasoactive intestinal polypeptide‐secreting tumour (VIPoma), adrenocorticotrophin‐secreting tumour (ACTHoma), Parathyroid hormone‐related peptide‐secreting tumour, calcitoninoma, growth hormone‐releasing hormone‐secreting tumour and somatostatinoma. The clinical features and biochemical parameters of these tumours have been extensively described.^12^

The palliative treatment of patients with metastatic or locally advanced GEP NET depends on several patient and tumour characteristics. These include tumour grade, tumour growth rate, hormonal production, primary tumour origin and localisation and extent of metastases. Surgical debulking with curative or palliative intent can be considered in all G1 and G2 GEP NET in the presence of locoregional or liver metastases. For most of the functioning syndromes, patients should generally be started on somatostatin analogues (SSA) as first‐line treatment. Antiproliferative systemic options for GEP NET include therapy with SSA, everolimus or in the case of pancreatic NET (panNET) sunitinib and systemic chemotherapy.^13^, ^14^, ^15^

Additionally, peptide receptor radionuclide therapy (PRRT) with [^177^Lu‐DOTA^0^,Tyr^3^]octreotate (^177^Lu‐DOTATATE) has been shown as an effective treatment option for patients with an advanced GEP NET.^13^, ^14^, ^15^ PRRT with ^177^Lutetium (^177^Lu)‐DOTATATE was approved by the European Medicines Agency in 2017 and by the US Food and Drug Administration (FDA) in 2018 for the treatment of somatostatin receptor (SSTR) positive GEP NET, including foregut, midgut and hindgut NET in adults after the positive results of the randomised phase III Neuroendocrine Tumours Therapy 1 (NETTER‐1) trial.^10^, ^16^, ^17^, ^18^, ^19^ In 2024, the FDA approved PRRT with ^177^Lu‐DOTATATE for paediatric patients 12 years and older with the same indications.^20^

PRRT constitutes a form of radionuclide therapy that has revolutionised oncological therapy and is currently being assessed for other cancer subtypes. Within the NET field, PRRT is an evolving therapy, with the potential for use for other indications than the current label of advanced G1 or G2 GEP NET. In this review, we provide an overview of the current clinical practice of PRRT in patients with GEP NET G1 and G2.

## 2 BACKGROUND AND CLINICAL DEVELOPMENT OF PRRT

**FIGURE 1:**
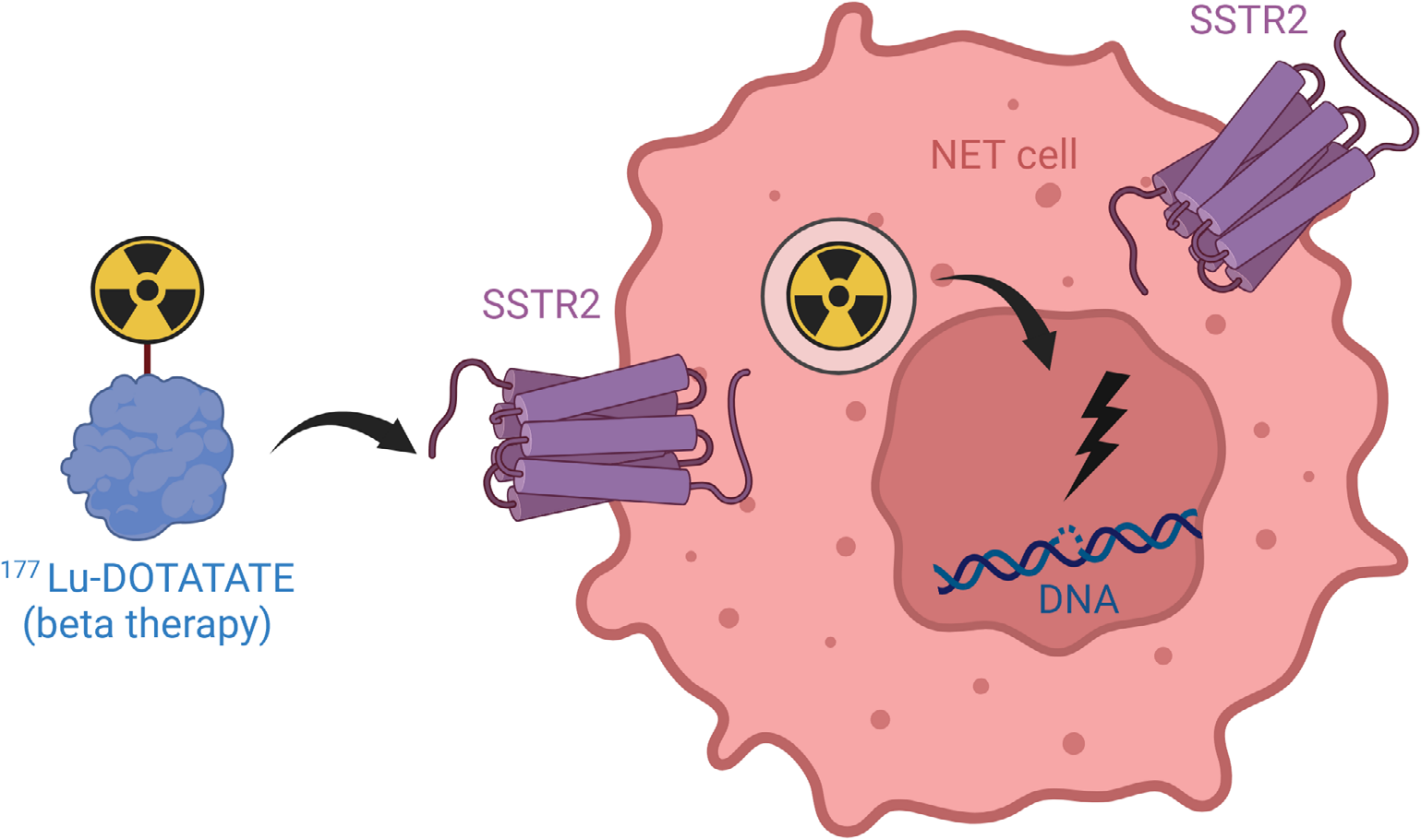
Mechanism of action of peptide receptor radionuclide therapy. After intravenous administration, [^177^Lu‐DOTA^0^,Tyr^3^]octreotate (^177^Lu‐DOTATATE) enters the NET cell by binding to the SSTR2. ^177^Lu beta‐particles induce local radiation resulting in DNA single strand breaks and DNA double strand breaks. Leading to tumour cell death induction. NET cell, neuroendocrine tumour cell; SSTR2, somatostatin receptor 2 (image created with BioRender.com accessed on 26 June 2024).

PRRT using ^111^Indium (^111^In)‐pentetreotide was first introduced into a NET patient at the Erasmus Medical Centre in 1994, following the advent of SSTR‐based molecular imaging with this radiolabelled SSA.^21^, ^22^, ^23^, ^24^, ^25^ PRRT acts by linking a radionuclide to a SSA, which can target the SSTRs on NET cells. SSTR comprise a group of G‐protein coupled receptors, of which the type 2 (SSTR2) is expressed in over 85% of NET.^26^, ^27^ After binding of the radiolabelled SSA to the SSTR2 this complex is internalised into the cell, where the emission of radiation locally causes DNA damage by irradiation. This exogenous damage consists of single‐ and double strand DNA breaks, which can overload the DNA repair mechanisms and subsequently lead to cell death (Figure 1).^28^, ^29^

During its clinical development, different combinations of SSA and radionuclides have been investigated for PRRT. The first combination used was pentetreotide coupled to ^111^In, which decays by electron capture with a half‐life of 2.8 days.^30^ It can be used for both diagnostic imaging and therapeutics, which is currently known as theranostics. Nonetheless, disease remission was rare after treatment with high‐dose ^111^In‐pentreotide, due to low SSTR2 affinity and the radiation properties of ^111^In.^30^, ^31^

Consequently, beta (β)‐particles with a higher energy were investigated, namely ^90^Yttrium (^90^Y) and ^177^Lu, to increase efficacy. Both radionuclides have been studied for PRRT, but there has been no direct, prospective comparison between ^90^Y‐DOTA‐octreotide (^90^Y‐DOTATOC) and ^177^Lu‐DOTATATE.^30^, ^32^ Nevertheless, the median progression free survival (PFS) and OS within the single arm studies appear higher for ^177^Lu‐DOTATATE compared to ^90^Y‐DOTATOC, with PFS ranging from 28 to 36 and OS from 36 to 63 months for ^177^Lu‐DOTATATE, versus PFS of 17–29 months and OS of 22–37 months for ^90^Y‐DOTATOC, respectively.^33^ Differences in characteristics of the radionuclides must be taken into account. Both the E_βmax_ and penetration range are higher for ^90^Y compared to ^177^Lu, respectively 2.27 versus 0.49 MeV and 11 versus 2 mm, which increases the likelihood of developing toxicity in healthy tissues. For example, after treatment with ^90^Y‐DOTATOC more severe kidney toxicity has been reported in comparison to ^177^Lu‐DOTATATE.^17^, ^34^ Contrarily, ^90^Y has theoretical advantages for larger tumour lesions (~5 cm) with more heterogeneous uptake due to its longer β‐particle path length, whereas ^177^Lu might potentially be better suited to smaller lesions (~0.2 cm).^35^, ^36^ Furthermore, the decay schemes differ between both radionuclides, as ^177^Lu does emit gamma photons at 208 (10%) and 113 keV (6%), respectively, while ^90^Y emit almost pure β^−^ particles.^36^ ^177^Lu‐DOTATATE posttherapy scans can be made with planar scintigraphy or single‐photon emission computed tomography (SPECT). ^90^Y‐DOTATOC posttherapy scans can be made with Positron Emission Tomography/computed tomography (CT) or bremsstrahlung SPECT/CT.^37^, ^38^ Phase III registration has thus far only been executed for ^177^Lu‐DOTATATE.

[DOTA^0^,Tyr^3^]octreotide (DOTATOC) is another SSA used in several centres and clinical trials for PRRT with ^177^Lu. The affinity of ^177^Lu‐DOTATATE for the SSTR2 receptor is nine times higher compared to ^177^Lu‐DOTATOC.^39^ ^177^Lu‐ DOTATOC has a lower uptake in tumours, resulting in a shorter mean residence time and lower absorbed tumour dose.^39^, ^40^ However, the whole‐body retention of ^177^Lu‐DOTATATE is higher, which potentially causes higher bone marrow toxicity.^36^, ^40^ PRRT with either ^177^Lu‐DOTATATE or ^177^Lu‐DOTATOC is effective. ^177^Lu‐DOTATATE seems to have a more favourable profile and was used in the NETTER‐1 trial.^41^ However, the COMPETE and COMPOSE trials currently are comparing ^177^Lu‐DOTATOC to other NET therapies.^42^

### 2.1 PRRT administration

The treatment with PRRT requires specialised handling of high energy radionuclides, including dedicated clinical facilities, permits, radiopharmacy and trained staff. The most commonly used PRRT with ^177^Lu‐DOTATATE protocol involves four cycles of 7.4 Gigabecquerel (GBq) (200 mCi) of ^177^Lu‐DOTATATE at eight weekly intervals, which was used in de NETTER‐1 trial.^17^, ^41^ The interval can be varied between 6 till 12 weeks and extended till 16 weeks in case of toxicity. In between cycles, follow‐up is needed to assess patients for adverse effects, which includes monitoring of full blood count, liver and renal function.^17^ Another radiolabelled SSA which is currently in phase III clinical trials is ^177^Lu‐edotreotide with a dosing regimen of four cycles every 3 months or six cycles every 6–8 weeks.^42^, ^43^, ^44^ Before treatment, patients must have stopped long‐acting SSA for 4–6 weeks and short‐acting SSA for 24 h, due to potential competitive SSTR2 binding of these drugs. SSA may be used in between cycles for symptomatic control of functioning syndromes. To reduce the occurrence of nephrotoxicity, an amino acid infusion consisting of Lysine and Arginine is started 30–60 min prior to PRRT administration. This infusion usually continues 4 h, but can be extended, depending on the patient's renal and heart function. Amino acid infusion is also crucial during ^90^Y‐DOTATOC therapy due to the higher risk of kidney toxicity as compared to the ^177^Lu‐DOTA‐SSA.^17^ At the time of patient discharge, patient radiation exposure levels are assessed, to provide personalised advice regarding radiation safety precautions at home and to avoid a radiation exposure to other healthy people, particularly children and pregnant women.^45^

After the administration of radioactivity, posttherapy scans including planar scintigraphy and/or SPECT–CT can be made to verify for successful uptake of the radiopharmaceutical and/or disease progression (e.g. new lesions) in between cycles.^46^

After completing all cycles, response evaluation is recommended at 2–3 and 6 months by cross‐sectional and/or functional imaging.^17^ During this period, pseudo‐progression may occur, where increased tumour size is observed which does not reflect the actual tumour response. This growth is likely caused by swelling and necrosis due to a local inflammatory reaction in response to PRRT.^47^ In the long term, the frequency of follow‐up is determined individually based on the therapy response, toxicity of the PRRT and tumour characteristics.

### 2.2 Selection of patients for PRRT

Due to its mechanism of action, NET patients should be carefully selected before they undergo PRRT. First, PRRT with ^177^Lu‐DOTATATE is only effective when all NET localisations exhibit sufficient expression of SSTR. Therefore, the presence of SSTR‐negative metastases is considered a contraindication for PRRT, unless they can be treated otherwise, for instance with locoregional therapy. To evaluate whether a patient is eligible for PRRT, functional imaging should be performed to assess the SSTR expression in vivo.^48^, ^49^ Currently, the most commonly used tracers are ^68^Ga‐DOTATOC and ^68^Ga‐DOTATATE, which can be used for Positron Emission Tomography imaging and combined with cross‐sectional imaging with CT or magnetic resonance imaging. Although there are slight differences in affinity profile and individual lesion detection, ^68^Ga‐DOTATOC and ^68^Ga‐DOTATATE can be used interchangeably in clinical practice for the purpose of selection of suitable patients for PRRT.^50^ To determine whether SSTR expression is sufficient, a semiquantitative scale can be used, which is adopted from the Krenning scale for ^111^In‐pentreotide imaging.^51^ This semiquantitative scale compares uptake between tumour lesions and liver/spleen, resulting in an objective point scale were 0 corresponds with no uptake till 4 where tumour uptake is greater than the spleen. For PRRT with ^177^Lu‐DOTATATE, a score of at least 3 (greater than the liver) is necessary at all locations. Preferably, pre‐treatment imaging could serve as a predictive tool for anticipating therapy outcomes in advance, which is currently being extensively investigated. Various variables, including different types of the standardised uptake value and tumour‐to‐blood ratio, appear to be predictive for therapy outcome.^52^, ^53^, ^54^, ^55^, ^56^, ^57^, ^58^, ^59^, ^60^, ^61^ Nonetheless, the clinical utilisation of these values remains limited due small sample sizes with heterogenous patients and doubts about the generalisability of cut‐off values between different imaging modalities.^62^

Treatment should be administered in accordance with FDA and European Medicines Agency protocol approvals.^17^, ^63^ Accordingly, it is advised to discuss patient eligibility in a multidisciplinary team in a specialised NET centre. Eligible patients are required to adhere to specific radiation safety guidelines during and after treatment with PRRT. Furthermore, a Karnofsky performance status greater than 50 is required. The onset of PRRT efficacy may span several weeks to months, and the treatment duration itself extends to 6 months. Consequently, patients undergoing PRRT should have an expected survival of at least 3 months. Pregnancy, ongoing lactation, insufficient bone marrow reserves, creatinine clearance <30 mL/min, severe hepatic impairment and severe cardiac impairment are other contraindications.^17^, ^18^, ^19^, ^63^

To further improve patient selection, several predictive markers and patient characteristics are associated with treatment outcome of PRRT with ^177^Lu‐DOTATATE. Notably, Karnofsky performance <70, elevated baseline chromogranin A (≥4× upper limit of normal) and pretreatment with multiple lines of chemotherapy are associated with worse PFS and OS.^23^, ^55^, ^64^, ^65^ Midgut NET patients tend to have longer PFS than panNET patients after PRRT with ^177^Lu‐DOTATATE.^55^ Additionally, patients with a higher total tumour volume experience worse OS.^55^ Specifically, those with a high liver tumour burden (>50%) and multiple bone metastases (>5) exhibit poorer OS. Larger target lesions (>30 mm diameter), elevated alkaline phosphatase and hypoalbuminaemia are linked to worse PFS.^55^, ^65^, ^66^, ^67^ Pre‐existing ascites or the development of interim ascites also negatively affect OS.^65^ However, no single characteristic or marker independently predicts treatment response with ^177^Lu‐DOTATATE. Hence, novel predictive tests were investigated. A scoring model, using normalised chromogranin A, previous chemotherapy and creatinine, was described to predict treatment failure at 1 year, PFS and OS with an area under the receiver operating characteristic curve of 0.816. Transcriptomic assessments like the PRRT prediction quotient (PPQ) and NETest provide accurate predictions of response (PPQ: 96% predictive accuracy and NETest: 93% diagnostic accuracy).^68^, ^69^, ^70^ Ongoing investigations are exploring predictive expression profiles involving both miRNA and mRNA.^71^ Considering these predictive markers and patient characteristics factors is essential for tailoring PRRT to individual patient benefit.

### 2.3 Adverse effects

Due to the physiological expression of SSTR in healthy tissues, such as liver, spleen, pituitary, salivary glands, thyroid, adrenals, kidney, prostate, pancreas, intestine and blood vessels, PRRT can lead to adverse effects in patients.^27^, ^72^ Frequent mild subacute side effects associated with PRRT include abdominal pain (26%), temporary hair loss (11%–60%) and fatigue (40%).^17^, ^24^, ^33^, ^41^, ^73^, ^74^

Most frequent serious adverse effects of PRRT with ^177^Lu‐DOTATATE are haematological toxicity, nephrotoxicity and liver toxicity.^17^, ^24^, ^41^, ^73^, ^74^ Approximately 4%–11% of patients experience grade 3 or 4 haematological toxicity. Platelet, white blood cell count and haemoglobin toxicity at grade 3 or 4 occurs in 2%–11%, 1%–9% and 3%–7% of patients, respectively.^41^, ^75^, ^76^, ^77^, ^78^, ^79^ However, this toxicity does not significantly increase the risk of infections and rarely results in bleeding complications.^75^, ^80^ Importantly, after a median of 4 years, 1%–2% of patients may develop myelodysplastic syndrome and 0.7%–1.1% may develop leukaemia as a consequence of PRRT with ^177^Lu‐DOTATATE.^74^, ^75^, ^81^, ^82^, ^83^

The renal expression of SSTR and the proximal tubular reabsorption of ^177^ Lu‐DOTATATE lead to high exposure of radiation to the kidneys and potential radiation‐induced nephrotoxicity. An intravenous infusion of positively charged amino acids, lysine and arginine, should be co‐administered to reduce the tubular reabsorption of ^177^Lu‐DOTATATE up to 40%, thereby minimising nephrotoxicity.^84^ Despite the amino acid infusion, acute nephrotoxicity still occurs in 1%–5% of patients. However, long term renal dysfunction is observed in only <1% of patients.^75^, ^82^, ^85^, ^86^ Unfortunately, concomitant administration of kidney‐protective amino acids causes nausea and vomiting in 25%–36% and 10% of patients, respectively, necessitating prophylactic and on‐demand anti‐emetic administration.^33^, ^77^ In the NETTER‐1 trial, nausea (59%) and vomiting (47%) were more frequently observed, potentially due to the use of VAMIN‐18 instead of Lysine‐Arginine, which is currently used in daily practice.^41^

Liver toxicity is a rare adverse effect, occurring in approximately 1%–4% of patients and depends highly on the liver tumour burden.^77^, ^79^, ^82^ Additionally, some studies show an increase in serum aminotransferases and bilirubin in 2%–3% and 7% of patients, respectively.^75^, ^76^ Together, short‐term haematological, renal and hepatic severe toxicities can be dose‐limiting during PRRT. Adverse events result in a submaximal activity of PRRT in 26% of patients.^87^ According to guidelines, it is recommended to administer half of the original activity of ^177^Lu‐DOTATATE during the subsequent treatment cycle when a grade 3 or 4 toxicity has subsided within 16 weeks. If the toxicity persists beyond 16 weeks or recurs after the half dose, PRRT should be discontinued.^19^, ^76^ Other reasons for submaximal activity are progressive disease during PRRT (6%), clinical deterioration (2%), patient request (2%), ileus (2%), infections (2%), cognitive deterioration (1%) and other adverse events (1%).

Less frequent side effects include oedema of brain metastasis, compression of the spinal cord or nerve root, bowel obstruction, frozen abdomen and hormonal crisis. Radiation‐induced damage by PRRT can induce temporary oedema and can cause compressive symptoms or complications. In cases where GEP NET have metastasised to the central nervous system or vertebral bones, patients may experience symptoms due to oedema of brain metastasis, compression of the spinal cord or nerve root.^88^, ^89^, ^90^ Prophylactic therapy with corticosteroids, administered after ^177^Lu‐DOTATATE infusion, can be considered to mitigate radiation‐induced oedema.^90^

Small intestinal NET (siNET) that metastasized to the local lymph nodes can give rise to mesenteric fibrosis, which is possibly mediated by local serotonin excess.^91^, ^92^ This fibrosis, characterised by the deposition of fibrous tissue in the mesentery, can lead to complications such as bowel obstruction and ischaemia.^92^, ^93^ Case reports and small series suggest that PRRT‐induced oedema can aggravate or elicit fibrosis‐related symptoms. Six percent of patients with a significant desmoplastic fibrosis tethering bowel can develop bowel obstruction within 3 months of treatment with ^177^Lu‐DOTATATE. In some cases, patients with peritoneal carcinomatosis experience a condition known as ‘frozen abdomen’, characterised by extensive adhesions between the bowel and the anterolateral abdominal wall, resulting in intestinal immobility. Administration of corticosteroids immediately post‐PRRT can be considered as a preventive measure.^93^

PRRT can result in excessive release of metabolically active amines or peptides from the tumour, leading to a hormonal crisis in approximately 1% of all patients and 9% of patients with a known hormonal syndrome.^94^, ^95^ Treatment of these crises is hormone‐specific, but often includes re‐introduction of SSA. SSA can safely be resumed 1 h after the infusion of ^177^Lu‐DOTATATE, to prevent interference of the SSTR occupancy.^96^

## 3 INDICATIONS FOR PRRT

### 3.1 Progressive, advanced GEP NET

The efficacy of PRRT in advanced, progressive, SSTR–positive midgut NET was confirmed with the NETTER‐1 trial. This international, multicentre, open‐label, randomised, phase III trial compared the intervention of ^177^Lu‐DOTATATE with 30 mg octreotide long‐acting repeatable (LAR) every 4 weeks with the control group high‐dose octreotide LAR 60 mg every 4 weeks. ^177^Lu‐DOTATATE was given at a dose of 4 times 7.4 GBq every 8 weeks. One hundred eleven patients were randomised in the ^177^Lu‐DOTATATE group and 110 in the control group. At primary analysis, the primary endpoint of median PFS was not reached in the ^177^Lu‐DOTATATE group and was 8.4 months in the octreotide LAR group with a significant hazard ratio (HR) of 0.21 in favour of ^177^Lu‐DOTATATE (*p* < .001). The response rates were 18% for patients receiving ^177^Lu‐DOTATATE and 3% for patients on octreotide LAR alone (*p* < .001). The rates of grade 3 or 4 overall adverse effects were similar between both groups. However, there was a grade 3 or 4 neutropenia, thrombocytopenia and lymphocytopenia reported in 1%, 2% and 9% of ^177^Lu‐DOTATATE patients, respectively, versus no patients in the control group.^41^ Median time to deterioration of QoL was also prolonged in the ^177^Lu‐DOTATATE group, compared to the control arm.^97^

During the final analysis of OS, 36% of the control group patients had crossed over to the ^177^Lu‐DOTATATE during follow‐up. Median OS as a secondary endpoint was not significantly improved at 48.0 months in the ^177^Lu‐DOTATATE group versus 36.3 months in the control group (HR 0.84, *p* = .30). During the long‐term follow‐up, two of 111 (2%) of patients in the ^177^Lu‐DOTATATE group developed myelodysplastic syndrome (Table 1).^82^

**TABLE 1:** Overview of the most relevant studies on peptide receptor radionuclide therapy in gastroenteropancreatic neuroendocrine tumour (GEP NET) with first‐ and second‐line.

Study	Number of patients	Study design	Setting	Median progression free survival (months)	Median overall survival (months)	Response rate (%)	Adverse effects grade 3 or 4 (%)	Haematological adverse effects grade 3 or 4 (%)
Strosberg et al.^41^, ^82^, ^97^	117	Randomised controlled trial, prospective	Midgut NET, G1–2, 2nd line	Estimated 20	48	18%	41%	9%
Singh et al.^99^	151	Randomised controlled trial, prospective	GEP NET, G2–3, 1st line	22.8	Not reached	43%	35%	14%
Brabander et al.^75^	443	Observational study, prospective	GEP and bronchial NET, G1–2	29	71 panNET 60 midgut NET	–	–	10%
Baum et al.^138^	378	Observational study, retrospective	NEN, G1–3	17	44	–	–	–
Mitjavila et al.^139^	522	Observational study, retrospective	NEN, G1–3	19.8 panNEN 31.3 midgut NEN 23.3 other GEP NEN	34.2 pan NEN 50.8 midgut NEN Not reached other GEP NEN	31.5% (GEP NEN)	4.7%	4.7%
Gordon et al.^140^	143	Observational study, retrospective	NEN, G1–3	32.3	72.5	–	–	‐
Tham et al.^79^	107	Observational study, retrospective	NEN, G1–3	54	Not reached	37.9%	–	9%

Abbreviations: G, grade; NEN, neuroendocrine neoplasm; NET, neuroendocrine tumour; panNET, pancreatic neuroendocrine tumour.

In the same year, a large single arm prospective cohort of 610 G1 and G2 GEP NET and bronchial NET patients treated with PRRT using ^177^Lu‐DOTATATE with a median follow‐up time of 64 months was analysed post‐hoc by Brabander et al. The dosing protocol was similar to the NETTER‐1 trial. Median PFS after PRRT was 30 months for both panNET and midgut NET patients. Median OS was 71 months for panNET patients and 60 months for midgut NET patients. PRRT‐associated toxicity was comparable to that observed within the NETTER‐1 trial (Table 1).^75^

A meta‐analysis conducted by Wang et al. compared 22 studies involving 1758 advanced NET patients treated with PRRT. Studies included were published up until April 2019. Response evaluation criteria in solid tumours (RECIST), RECIST 1.1 and Southwest Oncology Group criteria were used to evaluate treatment response. Studies used either ^177^Lu‐DOTATATE or ^177^Lu‐DOTATOC and a range of different activities and cycles of PRRT were used. Despite the heterogeneity between studies, the meta‐analysis revealed a pooled disease response rates (DRRs) of 25%–35% and a pooled disease control rates between 79% and 83% (Table 1).^98^

The NETTER‐1 phase III trial combined with several institutional phase II studies demonstrates that PRRT with ^177^Lu‐DOTATATE is an effective, tolerable and safe treatment for advanced G1 and G2 GEP NET. Both PFS and QoL improved with treatment with PRRT compared to high‐dose SSA. Moreover, the retrospective studies indicate that PRRT may also be efficacious in higher‐grade GEP NET and NEN with other origins. However, these patients constituted a minority of the study populations, and larger cohorts are necessary to validate these observations, preferably in controlled trials.

### 3.2 First‐line therapy

The NETTER‐2, an open‐label, randomised, parallel, superiority phase III study recently demonstrated the efficacy and safety of PRRT with ^177^Lu‐DOTATATE as a first‐line therapy in patients with intermediate to high‐grade GEP NET.^99^ Newly diagnosed patients with higher G2 (Ki67 ≥10% and ≤20%) or G3 (Ki67 >20% and ≤55%) SSTR‐positive GEP NET were included. Patients were 2:1 randomised to either four cycles of 7.4 GBq ^177^Lu‐DOTATATE with octreotide LAR 30 mg or the control group high‐dose octreotide LAR 60 mg every 4 weeks alone. The primary endpoint, PFS by RECIST 1.1 or death of any cause, was reached with a median PFS of 22.8 months in the ^177^Lu‐DOTATATE versus 8.5 months in the control group (*p* < .001).^99^ Objective response rate was significantly higher in the ^177^Lu‐DOTATATE group at 43.0% versus 9.3% in the control group. No new safety signals arose from this trial (Table 1).^99^ Consequently, the NETTER‐2 randomised trial shows superiority of PRRT over high‐dose SSA in first‐line setting of GEP NET with a Ki67 index between 10% and 55%.^99^ This illustrates its efficacy in GEP NET other than midgut origin and provides rationale for first‐line treatment in patients with a Ki67 index above 10%, which were not included in the randomised phase III trials of the SSA octreotide LAR and lanreotide autogel.^100^, ^101^ Still, additional research is required to conduct a comparative analysis between PRRT and other treatment modalities, such as targeted therapy or chemotherapy, in high‐grade GEP NET. An ongoing study, the COMPETE trial, is comparing first‐line treatments ^177^Lu‐Edotreotide versus everolimus in advanced GEP NET. The results of this comparative clinical trial are aimed at resolving this knowledge gap.^42^

### 3.3 Symptom control

Patients with NET have a worse QoL compared to the general population and other patients with cancer.^102^, ^103^ Partly, this can be caused by the occurrence of a NET‐associated functioning syndromes.^9^ PRRT has been shown to exert a positive effect on symptom control in advanced NET patients. QoL was measured 6 weeks, 3 and 6 months after PRRT with ^177^Lu‐DOTATATE in a prospective study of 265 advanced bronchial and GEP NET patients using the QoL Questionnaire‐Core 30 (QLQ‐C30). Global health status/QoL, insomnia, appetite loss and diarrhoea improved significantly, 36% (*p* < .01), 59% (*p* < .001), 63% (*p* < .01) and 67% (*p* < .001), respectively, after PRRT with ^177^Lu‐DOTATATE.^104^

QoL during PRRT with ^177^Lu‐DOTATATE was measured in another prospective study using the QLQ‐C30 and gastro‐intestinal NET (giNET) cancer‐specific module (QoL gastro‐intestinal NET 21). This study included 124 patients with siNET, 45 patients with panNET and 35 patients with other NET subtypes. QLQ‐C30 showed a global QoL improvement in NET patients following the fourth cycle of PRRT compared to baseline. Other improvements in QoL were observed in role and emotional functioning. Symptom relief was achieved concerning fatigue, nausea, vomiting, pain, insomnia, appetite loss and diarrhoea. Additionally, the QoL gastro‐intestinal NET 21 showed improvement in social functioning and a decrease of symptoms in flushing, night sweats, gastrointestinal symptoms, treatment‐related symptoms, disease related worries and weight loss. Patients with functioning tumours experienced a better improvement in symptoms compared to patients with non‐functioning tumours.^105^

Other studies have also shown the benefit of PRRT for patients suffering from metastatic functioning NET (Table 2). The effect of PRRT on refractory carcinoid syndrome symptoms was investigated in a retrospective analysis in 22 metastatic midgut NET patients without radiological progression at the start of PRRT with four cycles of ^177^Lu‐DOTATATE 7.4 GBq. Mean daily bowel movement frequency decreased from 6.1 to 4.6 after PRRT, while the mean number of daily flushes decreased from 4.3 to 2.4 after PRRT. This was accompanied by a decrease of median 24‐h u5‐HIAA excretion from 775 to 530 μmol/24 h (Table 2).^106^ Regarding patients with advanced insulinoma, Friebe et al. investigated hypoglycaemia before and after PRRT with ^90^Y‐DOTATOC and ^177^Lu‐DOTATOC, in 26 patients. A semiquantitative scoring system was used to quantify the severity and frequency of hypoglycaemic episodes, from score 0, no hypoglycaemic episodes to score 2, severe hypoglycaemia requiring hospitalisation and combined medication or history of hypoglycaemic coma. A total of 21 of 26 patients (81%) demonstrated improvement of hypoglycaemic score during long term follow‐up after PRRT. Average time of symptomatic benefit was 17.2 months. Five patients (19%) experienced transient hypoglycaemia after PRRT infusion (Table 2).^107^ Similar results of symptom control were seen in our five patients with advanced insulinomas with a history of severe, life‐threatening hypoglycaemia receiving ^177^Lu‐DOTATATE or ^111^In‐Octreotide.^108^

**TABLE 2:** Overview of the most relevant studies on peptide receptor radionuclide therapy in functional neuroendocrine tumour.

Study	Number of patients	Setting	Quality of life	Overall symptom improvement
Zandee et al.^106^	22	Advanced midgut NEN with refractory carcinoid syndrome	Not significantly improved	67%
Friebe et al.^107^	26	Metastatic NEN with Insulinoma	–	81%
Zandee et al.^95^	34	Metastatic functioning panNET	Improved significantly	71%
Bongiovanni et al.^109^	68	Advanced functioning NEN	–	91%

Abbrevaitions: NEN, neuroendocrine neoplasm; panNET, pancreatic neuroendocrine tumour.

In our retrospective study, we investigated the effects of four cycles of 7.4 GBq ^177^Lu‐DOTATATE in 34 patients with a metastatic functioning panNET with different syndromes. The patient group included 14 insulinomas, five VIPomas, seven gastrinomas and eight glucagonomas. A symptomatic response was seen in 17 of 23 patients with uncontrolled symptoms at baseline. Response consisted of a decrease of hypoglycaemic events (in 67%), diarrhoea (in 80%), pyrosis and diarrhoea (67%) and decrease of skin lesions (71%) and increase of weight (71%), in insulinomas, VIPomas, gastrinomas and glucagonomas, respectively. Patients experienced a significant improvement in QoL 3 months after the final PRRT (*p* = .002) (Table 2).^95^ A study by Bongiovanni et al. confirmed the effects of PRRT in metastatic functional NET. Sixty‐five functional NET patients, comprised of carcinoid syndrome (91%), insulinoma (7.4%) and ACTHoma (1.5%), were analysed. Patients received treatment with ^177^Lu‐DOTATATE with a medium cumulative activity of 22.2 GBq. Of the 65 patients, 59 showed syndrome response defined as, ≥30% reduction in frequency of bowel movements for carcinoid syndrome, normalisation of glycaemic level for insulinoma and normalisation of ACTH in ACTHoma. Median time to syndrome response was 5.0 months (Table 2).^109^ Together, these studies demonstrate that PRRT represents a therapeutic option for improving QoL and mitigating symptoms associated with various functioning NET. However, it is crucial to exercise caution due to the occurrence of hormonal crises following the infusion of ^177^Lu‐DOTATATE, such as carcinoid crisis or hypoglycaemia. Notably, patient sample sizes for specific syndromes remain limited, and not all hormonal syndromes have been thoroughly investigated.

As mentioned earlier, siNET patients can suffer from symptoms due to desmoplastic fibrosis within mesenteric lymph node metastases.^92^ We previously conducted a comprehensive retrospective study to investigate clinical behaviour of metastatic mesenteric masses in 530 siNET patients. Among these patients, 132 received PRRT. Only 3.8% of patients treated with PRRT experienced an objective size reduction in their mesenteric masses, while an overall objective response incorporating other target lesions was observed in 12.9%.^91^ Similar results were observed by Mansour et al., who conducted a retrospective study to access the impact of PRRT on 52 patients with a siNET G1 or G2 and a mesenterial mass. Following PRRT, overall response rate (ORR) of the mesenterial mass was 4% and for non‐mesenterial mass 8%. Mesenteric mass‐related symptoms were reduced in 46% of patients. In 12% of patients, long‐term gastrointestinal complications occurred.^110^ Evidence thus far suggests that PRRT lead to a reduction of siNET‐associated mesenterial mass in a very small subgroup of patients. However, PRRT could be considered for management of mesenteric mass‐related symptoms. Close monitoring for bowel obstruction is essential in patients with mesenteric mass undergoing PRRT. These findings emphasise the need for careful patient assessment and monitoring during PRRT, especially in cases where symptomatic mesenteric masses are present.

### 3.4 Induction PRRT

Given its potential in tumour reduction, PRRT has been studied as a downstaging therapy for panNET, which presented with locally advanced disease. Such a strategy could allow surgical resection or improve surgical outcomes in patients who were initially unfit to undergo curative surgery or in whom it was judged that they were at an unacceptable high risk of complications. We investigated the effects of preoperative downstaging PRRT for 49 locally advanced or oligometastatic panNET. Following PRRT with ^177^Lu‐DOTATATE, partial response according to RECIST 1.1 occurred in 45% of patients, while 38% of patients had downstaging of vascular involvement. Twenty‐six patients successfully underwent surgery after PRRT. Patients treated with PRRT, and surgery had a longer OS than patients treated with PRRT only, respectively 14.7 and 5.5 years (*p* = .003).^111^ The NeoLuPaNET trial investigators presented published their results recently. Following treatment with neo‐adjuvant PRRT, a partial response was observed in 18 of 31 panNET patients. In this study, 96.5% of patients could undergo surgery after neo‐adjuvant PRRT, where severe postoperative complications were observed in 24% of patients.^112^ Although the investigation of induction PRRT remains limited to small patient cohorts, the partial response observed, thus enhancing the feasibility of subsequent surgery and the potential for prolonging OS, holds promise as a therapeutic approach for patients with advanced NET. However, cautious consideration and thorough patient assessment is essential due to the regular occurrence of severe postoperative complications on a regular basis. Further research and well‐defined patient selection criteria are warranted to fully assess the efficacy and safety of induction PRRT.

## 4 PRRT IN CHALLENGING PATIENTS

Patients with GEP NET often present with characteristics that can complicate PRRT. Elderly patients, for example, form a challenging subgroup that might benefit from PRRT despite their complex clinical profiles. These patients typically have more comorbidities, lower performance scale, less bone marrow reserve and mild renal insufficiency. Although these factors alone may not contraindicate PRRT, their combination could necessitate dose adjustment and early evaluation. Advanced age is particularly associated with increased subacute haematological toxicity after PRRT, potentially requiring dose reduction.^78^ Patients with mild renal insufficiency, creatinine clearance between 30 and 50 mL/min, might benefit from PRRT, but it is crucial to prevent further decline in renal function. Although renal impairment after PRRT is rare, careful monitoring of renal function during and after PRRT is essential.^85^ Patients with extensive liver tumour burden (>50%) bone metastases, mesenterial mass or peritoneal disease may experience more side effects. Extensive liver tumour burden is linked to poorer OS and PFS compared to patients with lower liver tumour load.^65^, ^66^, ^78^, ^89^ Additionally, patients who have undergone multiple prior chemotherapy treatments generally have a worse OS, but can still benefit from PRRT.^64^, ^113^ Multidisciplinary discussion of all NET patients can aid in determining appropriate dose reductions, prophylactic medications and alternative treatment options.

### 4.1 ACCESSIBILITY AND COST‐EFFECTIVENESS OF PRRT

PRRT should preferably be administered in specialised NET centres equipped with a multidisciplinary team. However, such specialised care is not universally accessible, particularly in emerging and developing economies. A survey of Dureja et al. revealed that PRRT was only available to 37%–43% of patients. The affordability of PRRT was significantly lower in emerging and developing economies compared to advanced economies. Monitoring through conventional imaging was also less frequently affordable for patients in emerging and developing economies. Additionally, patients in these regions experienced fewer annual specialist visits, greater average travel distance and higher associated cost.^114^ A cost‐effectiveness study conducted in England demonstrated that PRRT is a cost‐effective treatment option for GEP NET compared to alternatives treatments.^115^ However, assessing cost‐effectiveness is inherently challenging, and extrapolating these findings to other countries is even more complex due to the variability in PRRT costs across different regions.^114^

## 5 SSTR ANTAGONISTS

Another therapeutic strategy for PRRT has been to utilise radiolabelled SSTR antagonists instead of SSTR agonists. SSTR antagonists have been shown to lead to higher tumour dose and tumour‐to‐organ ratios than agonists in pre‐clinical research, due to their capability to bind to the SSTR2 in its active and inactive states. An example of a radiolabelled SSTR antagonist is ^177^Lu‐satreotide. A phase I study evaluated the safety of ^177^Lu‐satreotide in 20 patients with metastatic NET. The expected dose was determined by dosimetry and the maximum allowable activity per administration was 7.4 GBq. Importantly, 57% of patients who received a second cycle developed grade 4 haematotoxicity. They had received a bone marrow dose of ≥1.44 Gy. After this finding, the study continued with half the dose of ^177^Lu‐ satreotide in the remaining patients. ORR was 45%, while median PFS was 21 months.^116^ Wild et al. investigated ^177^Lu‐satreotide in a phase I/II study with 40 NET patients, predominantly of GEP origin. Patients received 4.5 GBq or 6 GBq per cycle for a total of three cycles. The median cumulative activity was 13.0 GBq over three cycles. 42.5% of the patients experienced grade 3 or 4 adverse events. ORR was 21% and median PFS 28.1 months.^117^ Dosimetry study in the same population showed good tumour uptake and retention of ^177^Lu‐satreotide.^118^ ^177^Lu‐DOTA‐LM3 is another SSTR antagonist investigated in a study in 51 patients by Baum et al. The median activity per cycle in this study was 6.1 ± 0.8 GBq. Partial response was seen in 36.2% and stable disease in 48.9% of the patients. Grade 3 thrombocytopenia occurred in 5.9% and lymphocytopenia in 7.8%.^119^ These studies indicate that SSTR antagonists exhibit greater haematological toxicity than initially anticipated. Consequently, the practical benefits of using these agents fall short of expectations, and it is unlikely that SSTR antagonists will be implemented in routine clinical practice soon. Other contemporary studies are currently investigating new radionuclides, such as alpha‐emitting particles ^212^Pb and ^225^Ac and beta‐emitting particle ^161^Tb to increase efficacy of PRRT even further.^120^, ^121^ Moreover, ^161^Tb might be used as surrogate for ^177^Lu, due to similar characteristics such as chemical properties, half‐life and energy. This is likely required in the future to meet the rising request for PRRT and insufficient supply of ^177^Lu.^121^

### 5.1 PRRT with extended half‐life circulation

The biological half‐life of ^177^Lu‐DOTATATE in circulation is relatively short, potentially resulting in inadequate tumour uptake and retention. To address this limitation and improve the efficacy of PRRT, a modified octreotate conjugated with Evans blue, ^177^Lu‐DOTA‐EB‐TATE, was developed. This modification extends the plasma half‐life due to albumin binding. The reduction in blood clearance achieved by ^177^Lu‐DOTA‐EB‐TATE leads to higher tumour doses, enhanced uptake and improved retention. Consequently, this approach holds promise for more effective tumour control in patients with NET.^122^ A dose‐escalating study with ^177^Lu‐DOTA‐EB‐TATE was performed by Liu et al. in 32 NET patients, mainly comprised of GEP NET G1 and G2. Patients were divided in three groups that received three cycles of 1.17 ± 0.09, 1.89 ± 0.53 or 3.97 ± 0.84 GBq per cycle, respectively. Grade 3 haematotoxicity was dose‐dependent and occurred between 0% and 21% of patients. DRR was between 43% and 50% of patients.^123^ A study by Jiang et al. investigated the safety and efficacy of ^177^Lu‐DOTA‐EB‐TATE in 30 metastatic NET patients, mainly GEP NET G1 and G2. The median cumulative administered activity was 8.97 GBq with activity per cycle being 3.7 GBq. Grade 3 haematotoxicity occurred in 13.3% of patients. Response rate was 33.3% and median PFS 36 months.^124^ Despite promising theoretical considerations and the investigation of a small patient cohort, further research on ^177^Lu‐DOTA‐EB‐TATE is needed before it can be considered a viable and safe alternative to ^177^Lu‐DOTATATE. Further research might also focus on replacing SSA for other NET‐specific targets to treat patients with SSTR‐negative NET.

### 5.2 Intra‐arterial PRRT

Intra‐arterial administration of PRRT was first investigated with the intention of improving liver tumour dose in patients with extensive liver metastases, since intravenous administration of PRRT is less effective in patients with bulky liver metastases.^23^, ^66^ Intra‐arterial PRRT allows direct delivery of ^177^Lu‐DOTATATE to the tumour sites within the liver and bypasses the first pass‐effect. A dosimetry study in 48 patients observed that the tumour dose absorbed was 62.2% higher with intra‐arterial PRRT compared to intravenous infusion of PRRT of ^177^Lu‐DOTATATE. The doses in the spleen and bone marrow were decreased with 30.7% and 37.5%, while the dose in the liver and lungs were increased with 40% and 8%.^125^ A study by Thakral et al. investigated the effect of intra‐arterial PRRT with dosimetry in 29 G1 and G2 GEP NET patients. The doses in the tumours were significantly higher in the intra‐arterial PRRT of ^177^Lu‐DOTATATE group with a dose per unit activity of 4.2 versus 2.68 mGy/MBq. The doses in the spleen and kidney did not differ significantly.^126^ Lawhn‐Heath et al. investigated the effect of intra‐arterial ^90^Y‐DOTATOC. This study was terminated after the first 10 patients due to lack of efficacy. Stable disease was observed in seven of 10 patients as the best response, whereas two patients showed progressive disease. Moreover, hepatic metastases did not have a higher uptake when ^90^Y‐DOTATOC was delivered intra‐arterially.^127^ The LUTIA trial investigated the benefit of intra‐arterial ^177^Lu‐DOTATATE in 27 patients with G1 and G2 GEP NET and bi‐lobar liver metastases. ^177^Lu‐DOTATATE was injected in either the left or right hepatic artery and while the contralateral hepatic lobe was used as intravenous control after the first pass. Tumour to non‐tumour uptake was compared in tumour lesions from both liver lobes. In the lesions treated through intra‐arterial injection this ratio was 17.9, while this was not significantly different compared to the lesions in the control lobe. Partial response rate was seen in 25% of patients, with no differences in intra‐arterial treated or control lobes.^128^ In summary, intra‐arterial PRRT does not appear to confer significant benefits compared to intravenous PRRT. Moreover, intra‐arterial PRRT constitutes a more invasive, expensive and time‐consuming procedure than intravenous PRRT, while there is a potential for increased morbidity associated with the intra‐arterial approach.

## 6 COMPARISON WITH OTHER SYSTEMIC THERAPIES IN GEP NET

The NETTER randomised trials have compared PRRT to a control arm with high‐dose SSA, to which it was superior in efficacy. Currently, there are no published comparative trails that randomise between PRRT and other systemic options, like chemotherapy or targeted therapy with everolimus and sunitinib. Everolimus is an oral inhibitor of mammalian target of rapamycin and registered for NET patients based on the RADIANT‐3 and RADIANT‐4 trials, showing a median PFS of 11 months in advanced, progressive panNET and non‐functioning giNET and bronchial NET patients.^129^, ^130^ Sunitinib is a multi‐targeted tyrosine kinase inhibitor, registered for G1 and G2 advanced, progressive panNET patients. In a phase III clinical trial an improved median PFS of 11.4 months in patients randomised to sunitinib was demonstrated compared with 5.5 months in patients randomised to placebo.^131^, ^132^ Sunitinib treatment was also associated with a significant improvement in OS (HR 0.41, *p* = .02).^131^ A direct comparison between sunitinib and PRRT was conducted in the OCLURANDOM trial, a multicentre randomised phase II trial, of which the results were presented in 2022. Patients with SSTR‐positive progressive advanced panNET were randomised between ^177^Lu‐DOTATATE (7.4GBq four times every 8 weeks) or sunitinib (37.5 mg a day). The primary endpoint of a minimum PFS rate difference of 25% at 12 months was met with 80.5% in the PRRT arm and 42% in the sunitinib arm. Median PFS in the PRRT arm was 20.7 months versus 11 months in the sunitinib arm. Grade 3–4 adverse events were 44% in the PRRT arm versus 60% in the sunitinib arm.^133^ These phase II randomised results demonstrate that PRRT is a more effective second line treatment with less toxicity than sunitinib.

Cabozantinib is another multi‐targeted tyrosine kinase inhibitor that was recently studied in the phase 3 CABINET trial. The CABINET trial compared cabozantinib versus placebo in patients with advanced NET G1‐G3 after progression on PRRT or targeted therapy. Cabozantinib significantly improved median PFS as compared with placebo, 8.4 months versus 3.9 months (HR 0.38, *p* < .001) in the extrapancreatic NET cohort and 13.8 months versus 4.4 months (HR 0.23, *p* < .001) in the panNET cohort, respectively.^134^ A direct comparison between PRRT and cabozantinib has not been performed. Nevertheless, cabozantinib should be considered as a next‐line treatment for progressive G1–3 GEP NET patients previously treated with PRRT.

Chemotherapy is another second‐line therapy for panNET. Although multiple regimens have been proposed in the past,^135^ best contemporary evidence for the efficacy of chemotherapy was shown in an open‐label, multicentre, randomised phase II trial comparing temozolomide alone versus capecitabine and temozolomide. The median PFS was significantly lower in the temozolomide group (14.4 months) than in the capecitabine and temozolomide group (22.7 months), while the DRR in the combination group was 39.7%. These results support capecitabine and temozolomide as a viable chemotherapeutic option for intermediate to high‐grade panNET.^136^ A direct comparison of second‐line therapies PRRT, targeted therapy and chemotherapy for advanced entropancreatic NET G1–3 patients was analysed in a multicentre, retrospective cohort study of 508 patients. The primary endpoint median PFS was significantly longer in the PRRT group compared with those in the chemotherapy or targeted therapy group, 2.2 years versus 0.6 years HR, 0.37 (*p* < .001), respectively.^137^ Prospective randomised direct comparison studies are currently ongoing. The COMPOSE trial will compare PRRT to standard of care, comprised of either chemotherapy or everolimus, as first‐line and second‐line therapies in high G2 and G3 GEP NET (ki67 index 15%–55%).^43^ The COMPETE trial is poised to set the standard for the second‐line treatment in advanced GEP NET, comparing ^177^Lu‐Edotreotide versus everolimus.^42^

## 7 PROPOSED USE OF PRRT

**FIGURE 2:**
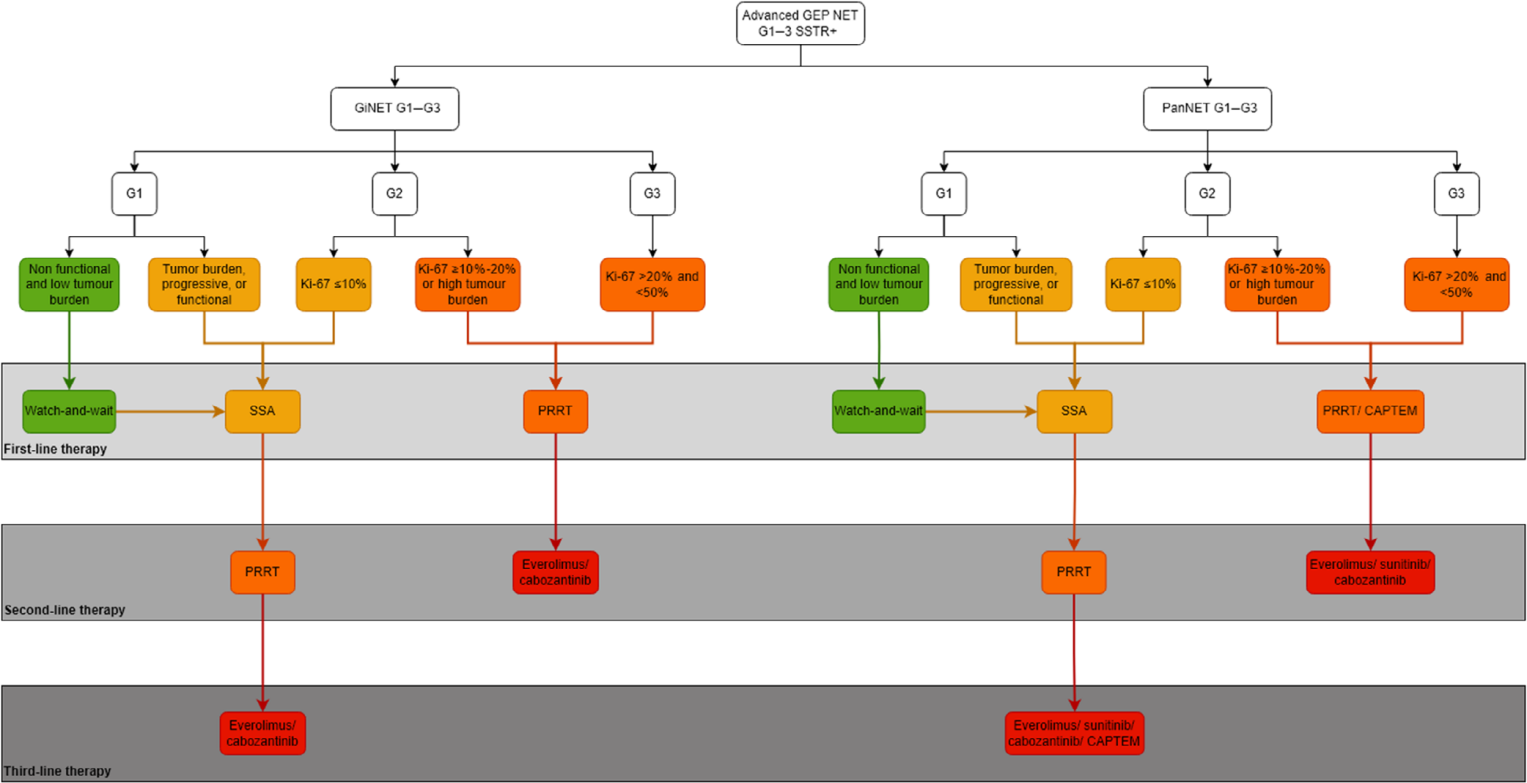
Flowchart of the proposed therapeutic algorithm of peptide receptor radionuclide therapy in grade 1–3 GEP NET. CAPTEM, capecitabine and temozolomide; G, grade; GEP NET, gastroenteropancreatic neuroendocrine tumour; giNET, gastro‐intestinal neuroendocrine tumour; panNET, pancreatic neuroendocrine tumour; PRRT, peptide receptor radionuclide therapy; SSA, somatostatin analogues.

The current guidelines from ENETS, ESMO and ASCO recommend that PRRT is suitable for treating G1–3, SSTR‐positive advanced GEP NET as a second‐ or third‐line treatment.^13^, ^14^, ^15^ The specific role of PRRT varies according to the grade and type of GEP NET, as outlined in each guideline. SSA are the first‐line therapy for G1 or low Ki‐67 index (≤10%) G2 GEP NET with stable disease or slow growth. The ENETS guideline also suggests a watch‐and‐wait approach for low grade and non‐functional GEP NET.^13^ Upon progression on SSA, PRRT becomes the preferred treatment for giNET. For panNET G1, or low Ki‐67 index (≤10%) G2 PRRT, chemotherapy, everolimus or sunitinib are all second‐line treatment options. For high Ki‐67 (>10%) G2 panNET with stable disease or slow growth and G3 GEP NET chemotherapy is first‐line therapy, with PRRT, everolimus, sunitinib and cabozantinib are second‐line options.^13^, ^14^, ^15^ However, based on the recent findings from the NETTER‐2 trial, long‐term outcomes from the NETTER‐1 trial and the OCLURANDOM trial, a revised therapeutic algorithm for PRRT is proposed (Figure 2).^41^, ^82^, ^99^ PRRT should be considered a first‐line treatment for G2 GEP NET with a Ki‐67 ≥10%–20% or high tumour load and for G3 GEP NET Ki‐67 >20% and <50%.^99^ Additionally, PRRT should be the second‐line therapy of choice for both G1 and low Ki‐67 index (≤10%) G2 GEP NET with stable disease of slow growth, as supported by the OCLURANDOM trial.^133^ Following PRRT, everolimus and cabozantinib should be administered for giNET, while CAPTEM, everolimus, sunitinib and cabozantinib should be considered for panNET. The results of the COMPOSE and COMPETE trials are anticipated to provide further insights into the role of PRRT as a first‐line or second‐line treatment for G1–3 GEP NET.^42^, ^43^ Additional prospective clinical trials are necessary to explore other forms of PRRT including the use of alternative SSA, new radionuclides or even combination therapies.

## 8 CONCLUSION

PRRT is an effective and safe treatment for patients with advanced GEP NET G1, G2 and G3. Although PRRT has been used since 1992 and registered for therapy in advanced G1 and G2 GEP NET since 2017, the technique and treatment options keep evolving. Randomised controlled trials comparing PRRT with other systemic therapies are needed to solidify the role of PRRT in the treatment landscape of GEP NET. The indications for PRRT are expanding into first‐line and G3 GEP NET. Different treatment options with PRRT are currently investigated to further improve the QoL and OS of NET patients. The current developments might lead to an even more prominent role of PRRT in the treatment of GEP NET G1, G2 and G3 in the future.

## AUTHOR CONTRIBUTIONS

**Jelka Kuiper:** Conceptualization; writing – review and editing; writing – original draft. **Eline Zoetelief:** Conceptualization; writing – review and editing. **Tessa Brabander:** Writing – review and editing; supervision. **Wouter W. de Herder:** Writing – review and editing; supervision. **Johannes Hofland:** Conceptualization; writing – review and editing; supervision.

## CONFLICT OF INTEREST STATEMENT

Tessa Brabander has received speaker fees and research support from AAA/Novartis. Wouter W de Herder reports consultancy, advisory roles, honoraria from Ipsen, Novartis, Camurus; support for travel/accommodation from Ipsen. Johannes Hofland has received speaker and/or consultancy fees from Ipsen, Serb and Novartis.

### PEER REVIEW

The peer review history for this article is available at https://www.webofscience.com/api/gateway/wos/peer-review/10.1111/jne.13469.

## Data Availability

Data sharing not applicable to this article as no datasets were generated or analysed during the current study.
